# Latent profile analysis of medication adherence in lower extremity deep venous thrombosis-cross-sectional study

**DOI:** 10.1371/journal.pone.0340406

**Published:** 2026-01-20

**Authors:** Ningning Hu, Xiaoyan Li, Feng Fu, Linzhou Xie, Jinfang Qi, Ping Wu, Yufeng Li, Ying-lan Li

**Affiliations:** 1 School of Nursing, Xinjiang Medical University, Urumqi, China; 2 The Sixth Affiliated Hospital of Xinjiang Medical University, Urumqi, China; 3 The Second Affiliated Hospital of Xinjiang Medical University, Urumqi, China; 4 Teaching and Research Section of Clinical Nursing, Xiangya Hospital, Central South University, Changsha, China; 5 Health Care Research Center for Xinjiang Regional Population, Urumqi, China; Celal Bayar University: Manisa Celal Bayar Universitesi, TÜRKIYE

## Abstract

**Background:**

The cornerstone of treating lower extremity deep venous thrombosis (LEDVT) lies in anticoagulation therapy to prevent thrombus progression and recurrence. However, patient adherence to medication is a critical factor influencing treatment efficacy. Traditional research often simplifies adherence into binary categories of “adherent” and “non-adherent,” which fails to comprehensively reflect the complex behavioral patterns. Based on latent profile analysis (LPA), medication adherence in LEDVT patients can be categorized into distinct classes, enabling more precise identification of their characteristics. Therefore, exploring these latent classes and their influencing factors holds significant importance for optimizing intervention strategies and improving prognosis.

**Methods:**

A cross-sectional survey was used to study LEDVT. From March 14, 2024 to September 20, 2024, a random sampling method was used to recruit 469 patients with LEDVT from four grade-A tertiary hospitals in Urumqi, China. Participants completed questionnaires on general demographic information, the Medication Adherence Scale, the Perceived Health Competence Scale, the Herth Hope Index, the Patient Activation Measure, the Beliefs about Medicines Questionnaire-Specific. LPA was conducted to analyze the medication adherence characteristics of patients with LEDVT. Univariate analysis and multivariate logistic regression were used to identify the influencing factors of their latent profiles. Data analysis was performed using Mplus 8.3 and SPSS 25.0 software.

**Results:**

LPA was employed to investigate medication adherence in LEDVT patients, revealing three distinct latent classes: the poorest adherence group (44.99%), the moderate adherence group (19.83%), and the good adherence group (35.18%). The logistic regression results demonstrated that, perceived health competence, hope, activation, beliefs about medication necessity, and concerns about medication were influential factors affecting the potential profile of medication adherence (all *p* < 0.05).

**Conclusions:**

LEDVT patients exhibit significant individual differences in medication adherence. Personalized intervention strategies can be designed based on different adherence classes to enhance medication adherence. Additionally, targeted interventions addressing perceived health competence, hope, positive affect, and medication beliefs can effectively improve adherence.

## Introduction

Deep Vein Thrombosis (DVT) refers to a pathological condition in which blood abnormally coagulates within the deep venous system, forming thrombi. Its pathogenesis is typically associated with Virchow’s triad, including hypercoagulability, endothelial injury, and venous stasis [1]. Among the various types of DVT, lower extremity deep vein thrombosis (LEDVT) is the most common. Mild cases may present with symptoms such as limb swelling, pain, and localized warmth, while severe cases may lead to pulmonary embolism (PTE) due to thrombus dislodgement, causing dyspnea, hypoxemia, and even life-threatening complications [2]. Additionally, organized thrombi may result in narrowing of the deep venous lumen, and prolonged venous stasis can increase venous pressure, causing irreversible damage to venous valves. This can lead to post-thrombotic syndrome (PTS), a chronic venous insufficiency disorder [3]. The typical manifestations of PTS include persistent limb edema, skin pigmentation, pain, and chronic ulcers, which not only significantly reduce patients’ quality of life but also increase long-term healthcare burdens [4]. Therefore, the hazards of DVT are not limited to the acute phase, and its potential long-term consequences emphasize the importance of early diagnosis and active intervention to prevent the occurrence and progression of complications.

LEDVT is a common vascular disorder, and its treatment goals include preventing thrombus progression, reducing the risk of recurrence, minimizing the likelihood of PTE, and mitigating the occurrence of PTS [5]. Anticoagulant therapy is the cornerstone of DVT management. Commonly used anticoagulants include low-molecular-weight heparin (LMWH), direct oral anticoagulants (DOACs) such as rivaroxaban and apixaban, and vitamin K antagonists (e.g., warfarin) [6]. These agents effectively reduce thrombus-related complications by inhibiting the coagulation process [7]. However, the efficacy of anticoagulant therapy largely depends on patient adherence. Strict compliance with prescribed anticoagulant regimens, regular ultrasound examinations, and blood monitoring (e.g., INR monitoring for warfarin users) are critical to ensuring therapeutic efficacy and safety [8]. Poor adherence may lead to treatment failure, increasing the risk of thrombus recurrence, PTE and PTS [9]. Studies have shown that only 37.7% of patients strictly adhere to anticoagulant prescriptions [10], highlighting the widespread and urgent need to address patient adherence issues in clinical practice.

Medication adherence refers to the extent to which patients strictly follow the prescribed treatment regimen as recommended by healthcare professionals, including taking medications on time, in the correct dosage, and in the proper manner [11]. Studies have shown that good adherence to anticoagulant therapy not only effectively prevents thrombus reformation and recurrence but also significantly enhances treatment efficacy and safety [12]. However, the factors influencing medication adherence are multifaceted. They are not only related to patients’ own health competence but are also affected by their psychological state. For example, a patient’s ability to manage their health (perceived health competence), optimistic expectations regarding treatment outcomes (hope level), proactivity in coping with illness (activation), and beliefs about the necessity and side effects of medication (medication beliefs) are all considered key variables that significantly impact medication adherence. [13]. Currently, medication management for patients with LEDVT often lacks specificity. Interventions typically focus on generalized health education and reminder services, neglecting the heterogeneity among patients in terms of age, abilities, and psychological states. This “one-size-fits-all” management model fails to adequately address patients’ individualized needs [14].

Latent profile analysis (LPA) is a statistical method that categorizes study subjects into several latent classes based on observed manifest characteristics. Its primary aim is to identify the latent classification traits of individuals within a population and reveal the heterogeneity within the group [15]. Compared to traditional classification methods (e.g., cluster analysis), LPA places greater emphasis on uncovering the latent attributes of individuals while also generating probability distributions for the classes, significantly enhancing the interpretability and flexibility of the classification results [16]. As a crucial tool for studying population heterogeneity, LPA has been widely applied in fields such as social sciences, education, and medicine [17,18]. In the medical field, LPA is frequently used to analyze patient behavioral characteristics, treatment adherence, and other factors. Through this method, researchers can design more targeted intervention measures, improve patient management outcomes, and provide more precise theoretical support and data references for clinical decision-making.

Based on this, the present study employed LPA to classify medication adherence patterns among patients with LEDVT, and further explored the influencing factors associated with different adherence categories. By recognizing the characteristics and needs of different patient groups, this study aims to provide a basis for developing personalized intervention strategies, thereby optimizing disease management and potentially improving overall treatment outcomes and prognosis in patients with LEDVT.

## Methods

### Study design

This study adopts a cross-sectional research design for investigation and is designed and reported in accordance with the guidelines for epidemiological observational studies.

### Participants

From March 14, 2024 to September 20, 2024, patients with LEDVT were selected from four tertiary Grade A hospitals in Urumqi, Xinjiang as the study subjects. The selection of participants was based on the following criteria: (a) DVT event that developed after the patient’s primary hip or knee replacement surgery rather than the patient’s first DVT in their lifetime; (b) confirmed diagnosis of LEDVT by ultrasound; (c) currently taking rivaroxaban for anticoagulation. Exclusion criteria included: (a) concurrent life-threatening diseases, such as severe postoperative infection, PTE, or acute myocardial infarction; (b) inability to complete the questionnaire due to other reasons, such as severe cognitive impairment, vision or hearing deficits that prevent reading or comprehending the questionnaire, or severe mental illness.

A random sampling method was employed to select four districts from the seven administrative districts of Urumqi (Xinshi District, Toutunhe District, Shuimogou District, Dabancheng District, Shayibake District, Midong District, and Tianshan District). The selected districts were Xinshi, Shuimogou, Shayibake, and Tianshan. Subsequently, one Grade-A tertiary hospital was randomly chosen from each of the selected districts as the sample source unit. Within each hospital, LEDVT patients who met the inclusion and exclusion criteria were screened and selected as study participants.

### Sample size

The sample size was estimated using the following formula: N=[t_α_^2^P(1-P)]/d^2^,where N represents the required sample size for patients with LEDVT. In this formula, α = 0.05,t_α_ = 1.96,d = 0.05,P = 31.3% [19]. Considering potential issues with invalid questionnaires and sample loss during collection, the sample size was increased by 20%, resulting in an estimated minimum sample size of 414 cases. Moreover, following the recommendation, this study used the LPA method to ensure a sample size of 300–500 participants [20]. Ultimately, 469 LEDVT patients were included.

### Measurement

#### General demographic information.

The questionnaire included the following variables: (1) gender, (2) age, (3) accessibility of medication, (4) employment status, (5) presence of bleeding, (6) history of DVT, and (7) type of medication used. In this study, accessibility of medication refers specifically to the geographic convenience of obtaining rivaroxaban, such as the distance from the patient’s home to the hospital or pharmacy. History of DVT refers to any previous DVT episodes caused by other reasons prior to the current postoperative event. Type of medication refers to the number of different medications the patient was taking: “one type” indicates patients taking rivaroxaban only, whereas “two or more types” indicates patients taking rivaroxaban in combination with other long-term medications (e.g., antihypertensive or antidiabetic drugs).

#### Medication Adherence Scale, MAS.

The scale was developed by Ueno et al. [21]in 2018 and was later translated and adapted into Chinese by Zhang Lixiang et al. [22].This scale consists of four dimensions with 12 items, scored using a 5-point Likert scale ranging from 1 point for “never” to 5 points for “always.” The total score ranges from 12 to 60, with higher scores indicating a higher level of medication adherence. The Cronbach’s α coefficient of the scale was 0.802. The MAS was used to assess patients’ adherence to their rivaroxaban anticoagulant therapy.

#### Perceived Health Competence Scale,PHCS.

The scale was developed by Smith et al. [23] in 1995 and later translated and adapted into Chinese by Liu Hongxia et al. [24]. This questionnaire consists of two dimensions with eight items, scored using a 5-point Likert scale ranging from 1 point for “strongly disagree” to 5 points for “strongly agree.” Items 1, 2, 6, and 7 are reverse scored. The total score ranges from 8 to 40, with higher scores indicating a greater perceived health capacity. The Cronbach’s α coefficient of the scale was 0.802.

#### Herth Hope Index, HHI.

The scale was developed by Herth et al. [25] in 1991 and later translated and adapted into Chinese by Wang Yanhua [26]. This scale consists of three dimensions with 12 items, scored using a 4-point Likert scale ranging from 1 point for “strongly disagree” to 4 points for “strongly agree.” The total score ranges from 12 to 48, with higher scores indicating a higher level of hope. The Cronbach’s α coefficient of the scale was 0.97.

#### Patient Activation Measure,PAM.

The scale was originally developed by Hibbard et al. [27] in 2004 and later refined by Hibbard et al. [28] in 2005,reducing the original 22-item scale to a 13-item version. It was subsequently translated and adapted into Chinese by Hong Yang et al. [29]. This scale consists of four dimensions with 13 items, scored using a 5-point Likert scale ranging from 0 points for “not applicable” to 4 points for “strongly agree.” The total score ranges from 0 to 52, with higher scores indicating a greater level of individual activation. The Cronbach’s α coefficient of the scale was 0.835.

#### Beliefs about Medicines Questionnaire-Specific, BMQ-Specific.

The scale was developed by Horner et al. [30] in 1999 and later translated and adapted into Chinese by Si Zaixia et al. [31]. This questionnaire comprises two dimensions: beliefs about the necessity of medication and concerns about medication, with a total of 10 items. It is scored using a 5-point Likert scale, ranging from 1 point for “strongly disagree” to 5 points for “strongly agree.” The total score for each dimension ranges from 5 to 25, with higher scores indicating stronger beliefs in the respective dimension. The Cronbach’s α coefficient of the questionnaire was 0.77.

### Ethical consideration

Ethical approval for this study was obtained from the Ethics Committee of Xinjiang Medical University (Approval No. XJYKDXR20240314062). All participants voluntarily took part in the study and completed the questionnaire after providing written informed consent. The research was conducted in full compliance with the principles outlined in the Declaration of Helsinki.

### Data analysis

Latent profile analysis was conducted using Mplus 8.3 to explore subgroups of medication adherence based on responses to each item. Model fitting and testing were performed sequentially from Model 1 to Model 4. Several information criteria were used to evaluate model precision, including the Akaike Information Criterion (AIC), Bayesian Information Criterion (BIC), Sample Size-Adjusted BIC (aBIC), and Entropy. Lower values of AIC, BIC, and aBIC indicated better model fit, while an Entropy value greater than 0.8 suggested a well-classified model. Additionally, model fit was assessed using the Lo-Mendell-Rubin likelihood ratio test (LMR) and the Bootstrapped Likelihood Ratio Test (BLRT), where a p-value of less than 0.05 was considered indicative of good model fit [32].

Statistical analyses were performed using SPSS 25.0.Categorical variables were described as frequencies and percentages and compared using the chi-square test. Continuous variables were tested for normality using the Shapiro-Wilk test, and all were non-normally distributed (p < 0.001); therefore, they were expressed as median (interquartile range), and intergroup comparisons were conducted using the Kruskal-Wallis H test. To adjust for baseline differences, Rank ANCOVA was performed to compare the adjusted differences among the three adherence classes in perceived health competence, hope, activation, belief in the necessity of medication and concern about medication, with age and accessibility of medication as covariates. Bonferroni-adjusted post-hoc comparisons were applied. To further identify independent predictors of adherence class membership, a multinomial logistic regression model was constructed using adherence class as the dependent variable, and variables with p < 0.05 in univariate analyses were entered as independent variables.

## Results

### Descriptive characteristics of the participants

A total of 697 patients diagnosed with LEDVT were initially screened. Of these, 228 were excluded for the following reasons: 157 did not meet the inclusion criteria, 47 declined to participate, and 24 had incomplete data. Consequently, 469 eligible patients were enrolled and completed the questionnaire, all of whom were included in the final analysis. The final sample consisted of 340 female participants (72.49%) and 129 male participants (27.51%). The detailed distribution is shown in Table 1.

**Table 1:** Characteristics of participant (*n* = 469).

Variable	Classification	*n*(%)
**Categorical variables**		
Gender	Male	129(27.51%)
	Female	340(72.49%)
Accessibility of medication	Inconvenience	212(45.20%)
	Convenience	257(54.80%)
Employment status	None	331(70.58%)
	Employed	34(7.25%)
	Retired (or Retiring)	104(22.17%)
Presence of bleeding	No	448(95.52%)
	Yes	21(4.48%)
History of deep vein thrombosis	No	460(98.08%)
	Yes	9(1.92%)
Type of medication	One type	258(55.01%)
	Two or more types	211(44.99%)
**Continuous variables**		***M* (*P***_**25**_**,*P***_**75**_)
Age (years)		61.00(57.00,68.00)
Medication adherence		35.00(24.00,43.50)
Perceived health competence		22.00(17.00,25.00)
Hope		29.00(25.00,32.00)
Activation		27.00(23.00,33.00)
Belief in the necessity of medication		13.00(10.00,16.00)
Concern about medication		14.00(13.00,16.00)

**Note:** The continuous variables listed in the table are non-normally distributed and are therefore presented as medians with interquartile ranges. The score ranges and interpretations for medication adherence, perceived health competence, hope, activation, beliefs in the necessity of medication, and concern about medication are described in detail in the measurement section.

### LPA of medication adherence in LEDVT

From Model 1 to Model 4, the twelve medication adherence items of patients with LEDVT were used as manifest variables for latent class modeling. Although the AIC values decreased progressively from Model 1 to Model 4, the BIC value increased instead of decreasing in Model 4. Additionally, the LMR value was greater than 0.05, leading to the exclusion of Model 4.Compared to Models 1 and 2, Model 3 exhibited the lowest AIC, BIC, and aBIC values, an entropy value >0.8, and significant LMR and BLRT values (<0.05). Moreover, each class contained a sufficient number of participants to meet the model-fitting requirements. Therefore, the three-class model was deemed the optimal solution (see Table 2).

**Table 2:** Model fit indices and evaluation for LPA of medication adherence in LEDVT (*n* = 469).

Model	Log (L)	AIC	BIC	aBIC	Entropy	LMR	BLRT	Class probability/%
1	−8440.059	16928.119	17027.733	16951.562	—	—	—	100
2	−6623.726	13321.451	13475.024	13357.593	0.984	<0.001	<0.001	47.12/52.88
3	−6432.294	12964.588	13172.119	13013.429	0.935	<0.001	<0.001	44.99/35.18/19.83
4	−6405.812	12937.625	13199.113	12999.164	0.885	0.9437	<0.001	44.99/17.70/11.51/25.80

Furthermore, based on the attribution probability matrix of the three-class latent model, the posterior probability mean values were all > 0.8, further confirming the reliability of the three-class model. Thus, Model 3 was selected as the final model (see Table 3).

**Table 3:** Probability matrix of three latent classes of medication adherence in LEDVT.

Category	Class 1	Class 2	Class 3
Class 1	0.999	0.000	0.001
Class 2	0.000	0.954	0.046
Class 3	0.011	0.047	0.943

**Note:** Class 1 was defined as the “Poor Adherence Group”, Class 2 was defined as the “Good Adherence Group”, Class 3 was defined as the “Moderate Adherence Group”.

**Fig 1:**
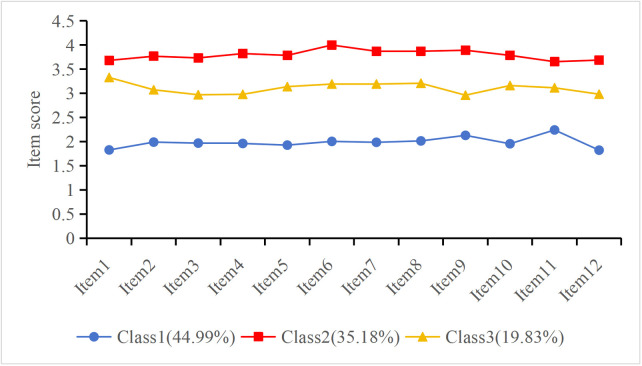
Three medication adherence Categories based on LPA.

LPA was conducted based on the item-level scores of the medication adherence scale, with higher scores indicating better adherence. As shown in Fig 1, the three patient classes exhibited distinct score patterns across the 12 items, ensuring a clear differentiation among low, moderate, and high levels of adherence. The scores of the three classes on the twelve medication adherence items are shown in Fig 1. Class 3, with moderate scores across all items, was named the “Moderate Adherence Group,” comprising 19.83% (n = 93) of the sample. Class 1, characterized by low scores across all adherence items, was designated as the “Poor Adherence Group,” accounting for 44.99% (n = 211) of the sample. Class 2, with relatively high adherence scores, was labeled the “Good Adherence Group,” representing 35.18% (n = 165) of the sample.

### Univariate analysis of potential categories of medication adherence

A univariate analysis was conducted using the three categories as the dependent variable. The results indicated that age, accessibility of medication, perceived health competence, hope, activation, belief in the necessity of medication and concern about medication showed statistically significant differences (*p* < 0.05). For detailed results, see Table 4.

**Table 4:** Univariate analysis of potential categories of medication adherence.

Variable	Participant	Class 1(n = 211)	Class 2(n = 165)	Class 3(n = 93)	*χ*^*2*^/*H*	*P*
**Categorical variables**						
Gender					3.900	0.142
male	129	66(31.28%)	44(26.67%)	19(20.43%)		
female	340	145(68.72%)	121(73.33%)	74(79.57%)		
Accessibility of medication					86.816	<0.001
Inconvenience	212	145(68.72%)	47(28.48%)	20(21.51%)		
Convenience	257	66(31.28%)	118(71.52%)	73(78.49%)		
Employment status					0.519	0.972
None	331	151(71.56%)	116(70.30%)	64(68.82%)		
Employed	34	15(7.11%)	11(6.67%)	8(8.60%)		
Retired (or Retiring)	104	45(21.33%)	38(23.03%)	21(22.58%)		
Presence of bleeding					5.213	0.074
No	448	206(97.63%)	153(92.73%)	89(95.70%)		
Yes	21	5(2.37%)	12(7.27%)	4(4.30%)		
History of deep vein thrombosis					3.519	0.172
No	460	208(98.58%)	163(98.79%)	89(95.70%)		
Yes	9	3(1.42%)	2(1.21%)	4(4.30%)		
Type of medication					0.032	0.984
One type	258	117(55.45%)	90(54.55%)	51(54.84%)		
Two or more types	211	94(44.55%)	75(45.45%)	42(45.16%)		
**Continuousvariables**						
Age (years)	469	62.00(58.00, 69.00)	60.00(57.00, 65.00)	62.00(58.00, 65.00)	9.173	0.010
Perceived health competence	469	17.00(15.00, 20.00)	25.00(23.00, 27.00)	23.00(21.00, 25.00)	250.394	<0.001
Hope	469	25.00(23.00, 27.00)	31.00(29.00, 34.00)	31.00(29.00, 33.00)	188.547	<0.001
Activation	469	24.00(21.00, 27.00)	30.00(25.00, 40.00)	34.00(31.00, 37.00)	171.335	<0.001
Belief in the necessity of medication	469	10.00(9.00, 12.00)	16.00(14.00, 17.00)	14.00(13.00, 15.00)	292.157	<0.001
Concern about medication	469	15.00(13.00, 17.00)	14.00(12.00,16.00)	14.00(12.00, 16.00)	8.045	0.018

**Note:** Class 1 was defined as the “Poor Adherence Group”, Class 2 was defined as the “Good

Adherence Group”, Class 3 was defined as the “Moderate Adherence Group”. Chi-square tests were used for categorical variables; Kruskal-Wallis H tests were used for continuous variables.

### Rank ANCOVA analysis

Before conducting the Rank ANCOVA, the scores of age, perceived health competence, hope, activation, belief in the necessity of medication, and concern about medication were rank-transformed. Age and accessibility of medication were included as covariates, with the five questionnaires as dependent variables and adherence class as a fixed factor in the Rank ANCOVA analysis. After adjusting for age and medication accessibility, the Rank ANCOVA results showed significant differences in the scores of perceived health competence, hope, activation, belief in the necessity of medication, and concern about medication among the three adherence groups (*p* < 0.05). Bonferroni post-hoc comparisons revealed the following, perceived health competence: significant differences were found between Class 1 and both Class 2 and Class 3 (*p* < 0.001).Hope: significant differences were observed between Class 1 and both Class 2 and Class 3 (*p* < 0.001), but no significant difference was found between Class 2 and Class 3 (*p* = 1.000).Activation: significant differences were found between Class 1 and both Class 2 and Class 3 (*p* < 0.001), and between Class 2 and Class 3 (*p* = 0.006).Belief in the necessity of medication: significant differences were found between Class 1 and both Class 2 and Class 3 (*p* < 0.001).Concern about medication: significant differences were observed between Class 1 and Class 2 (*p* = 0.034), while no significant differences were found between Class 1 and Class 3 or between Class 2 and Class 3 (*p* = 1.000).See Table 5.

**Table 5:** Results of rank ANCOVA for group comparisons on questionnaire scores, adjusted for age and availability of medication.

Variable	F	*p*	Post-Hoc comparisons (Bonferroni-corrected P-Value)		
			Class1 vs Class 2	Class1 vs Class 3	Class 2 vs Class 3
Perceived health competence	209.775	<0.001	<0.001	<0.001	<0.001
Hope	118.264	<0.001	<0.001	<0.001	1.000
Activation	106.325	<0.001	<0.001	<0.001	0.006
Belief in the necessity of medication	300.732	<0.001	<0.001	<0.001	<0.001
Concern about medication	3.461	0.032	0.034	0.235	1.000

**Note**: Class 1 was defined as the “Poor Adherence Group”, Class 2 was defined as the “Good

Adherence Group”, Class 3 was defined as the “Moderate Adherence Group”. The degrees of freedom for the overall model are (2, 464). The Bonferroni-adjusted post-hoc comparisons are reported for pairwise group differences.

### Factors influencing the three categories of medication adherence in LEDVT

Taking the three categories of medication adherence as the dependent variable (with the good medication adherence group as the reference). Variables that were statistically significant in the univariate analysis were included as independent variables. The parallel lines test showed that *p* < 0.001, indicating that the assumptions for ordinal regression were not met; therefore, so we constructed an unordered multi categorical logistic regression analysis. Collinearity diagnostics showed a tolerance <1 and a variance inflation factor (VIF) <10, indicating no collinearity issues. The logistic regression results demonstrated that perceived health competence, hope, activation, beliefs about medication necessity, and concerns about medication were influential factors affecting the potential profile of medication adherence (all *p* < 0.05). Specifically, compared with the good adherence group, patients with lower scores in perceived health competence, hope, activation, and belief in the necessity of medication were more likely to be classified into the poor adherence group, while patients with higher scores in concern about medication were also more likely to be classified into the poor adherence group. Compared with the good adherence group, patients with lower scores in perceived health competence and belief in the necessity of medication were more likely to be classified into the moderate adherence group. For detailed results, see Table 6.

**Table 6:** Multinomial logistic regression analysis of the three categories of medication adherence in LEDVT.

Variable	Class 1(ref. Class 2)					Class 3(ref. Class 2)				
	*β*	*SE*	Wald*χ*^2^	*P*	OR(95%CI)	*β*	*SE*	Wald*χ*^2^	*P*	OR(95%CI)
**Constant term**	35.262	5.221	45.619	<0.001	–	5.702	2.348	5.896	0.015	–
Age (years)	0.004	0.037	0.009	0.923	1.004(0.934 ~ 1.078)	0.032	0.018	3.229	0.072	1.033(0.997 ~ 1.070)
Accessibility of medication	0.883	0.572	2.385	0.122	2.418(0.789 ~ 7.416)	−0.579	0.345	2.810	0.094	0.561(0.285 ~ 1.103)
Perceived health competence	−0.356	0.107	11.010	0.001	0.700(0.567 ~ 0.864)	−0.135	0.058	5.399	0.020	0.874(0.780 ~ 0.979)
Hope	−0.356	0.080	19.750	<0.001	0.701(0.599 ~ 0.820)	−0.045	0.039	1.359	0.244	0.956(0.886 ~ 1.031)
Activation	−0.394	0.075	27.779	<0.001	0.674(0.583 ~ 0.781)	0.041	0.022	3.424	0.064	1.042(0.998 ~ 1.088)
Belief in the necessity of medication	−0.979	0.174	31.593	<0.001	0.376(0.267 ~ 0.528)	−0.346	0.087	15.943	<0.001	0.708(0.597 ~ 0.839)
Concern about medication	0.400	0.130	9.453	0.002	1.492(1.156 ~ 1.926)	0.026	0.066	0.152	0.697	1.026(0.902 ~ 1.167)

**Note:** Class 1 was defined as the “Poor Adherence Group”, Class 2 was defined as the “Good

Adherence Group”, Class 3 was defined as the “Moderate Adherence Group”.

## Discussion

In this study, LPA was used to classify medication adherence in patients with LEDVT. The results identified three distinct adherence categories: poor adherence, moderate adherence, and good adherence. This classification indicates significant heterogeneity in medication adherence among DVT patients, which is consistent with previous research [14]. Notably, the distribution among the three classes was unbalanced: the poor adherence group accounted for the highest proportion, at 44.99%, followed by the good medication adherence group at 35.18% and the moderate medication adherence group at 19.83%. This distribution suggests that nearly half of the patients exhibit poor medication adherence, underscoring the urgent need to develop and implement more targeted intervention strategies for this high-risk group to improve their medication adherence. The findings further demonstrate the important value of LPA in identifying underlying differences in patient adherence. This method effectively uncovers heterogeneity in medication behaviors that is often masked by traditional statistical approaches, thereby advancing adherence management from a “one-size-fits-all” model toward a more targeted and personalized approach.

This study found that perceived health competence was identified as a significant factor influencing the potential categories of medication adherence. Preliminary univariate analysis revealed significant differences in perceived health competence scores among the three groups (*p* < 0.001). The good adherence group (median: 25.00) and the moderate adherence group (median: 23.00) had higher scores than the poor adherence group (median: 17.00). After controlling for age and accessibility of medication, the Rank ANCOVA further confirmed these differences. Bonferroni post hoc tests indicated that not only were there significant differences between the poor adherence group and the other two groups (*p* < 0.001), but also between the good and moderate adherence groups (*p* < 0.001), suggesting a possible gradient relationship between perceived health competence and adherence level. Multinomial logistic regression analysis showed that, compared with the good adherence group, patients with lower perceived health competence scores were more likely to be classified into the poor adherence group (OR = 0.700, 95% CI: 0.567 ~ 0.864, *p* = 0.001) and the moderate adherence group (OR = 0.874, 95% CI: 0.780 ~ 0.979, *p* = 0.020), a result consistent with previous research [33]. Perceived health competence refers to an individual’s subjective assessment of their ability to manage their health, prevent diseases, and engage in health-promoting behaviors, serving as a fundamental basis for executing health-related behaviors [34]. Studies have shown that patients with higher perceived health competence typically exhibit stronger self-management awareness and initiative. They are more likely to actively seek health information, understand disease and medication-related knowledge, and have a greater sense of participation and control over their treatment [35]. This ability helps patients accurately perceive disease risks, enhance treatment confidence, and adopt proactive health behaviors, thereby promoting medication adherence to a certain extent. Notably, it plays a significant protective role, particularly among patients with lower adherence [36].

This study found that hope was a significant factor influencing the potential categories of medication adherence. Preliminary univariate analysis revealed significant differences in hope scores among the three groups (*p* < 0.001). The good adherence group and the moderate adherence group had the same median score (31.00), both higher than that of the poor adherence group (median: 25.00). After controlling for age and accessibility of medication, the Rank ANCOVA and Bonferroni post hoc comparisons further confirmed these differences: hope levels effectively distinguished the poor adherence group from the good and moderate adherence groups (*p* < 0.001), but there was no significant difference between the good and moderate adherence groups (*p* = 1.000). These findings suggest that maintaining an adequate level of hope is crucial for preventing poor medication adherence. Multinomial logistic regression analysis showed that, compared with the good adherence group, patients with lower hope scores were more likely to be classified into the poor adherence group (OR = 0.701, 95% CI: 0.599 ~ 0.820, *p* < 0.001), a finding consistent with previous research [37]. Hope is a positive psychological state characterized by an individual’s clear recognition of goals, strategic planning to achieve them, and confidence in their own abilities, leading to an expectation of favorable future outcomes [38]. Studies have shown that hope enhances psychological resilience, allowing patients to face disease and treatment with greater confidence and motivation, thereby actively participating in their health management [39]. For patients with LEDVT, the role of hope is particularly important. These patients require long-term anticoagulation therapy, and the side effects, uncertainties, and lifestyle inconveniences associated with treatment may weaken their adherence [40]. Hope can psychologically strengthen patients’ tolerance for treatment, stimulate their desire for health recovery, and consequently promote medication adherence [41].

This study found that activation was a significant factor influencing the potential categories of medication adherence. Preliminary univariate analysis revealed significant differences in activation scores among the three groups (*p* < 0.001). The good adherence group (median: 30.00) and the moderate adherence group (median: 34.00) both had higher scores than the poor adherence group (median: 24.00). This finding suggests that the relationship between activation and medication adherence may not be purely linear. Excessively high activation, if not accompanied by appropriate disease knowledge and self-management skills, may lead patients to make inappropriate self-adjustments to their medication regimen and deviate from medical advice, which could be one possible reason why adherence does not always reach an optimal level [42]. After controlling for age and accessibility of medication, the Rank ANCOVA and Bonferroni post hoc comparisons further confirmed these differences: significant differences were observed not only between the poor adherence group and the other two groups (*p* < 0.001) but also between the good and moderate adherence groups (*p* = 0.006). Multinomial logistic regression analysis showed that, compared with the good adherence group, patients with lower activation scores were more likely to be classified into the poor adherence group (OR = 0.674, 95% CI: 0.583 ~ 0.781, *p* < 0.001), a finding consistent with previous research [43]. Activation refers to an individual’s proactiveness, enthusiasm, and level of engagement when facing goals, tasks, or challenges. It reflects the willingness to exert effort and the presence of positive emotions in both behavioral and psychological aspects of goal attainment [44]. One possible explanation is that patients with higher activation levels tend to exhibit greater initiative and awareness of health management. They actively seek treatment-related knowledge and develop a deeper understanding of their disease and therapy, which in turn strengthens their confidence and motivation for treatment [45]. Additionally, highly activation patients are more likely to adopt proactive coping strategies when encountering side effects or treatment challenges, such as communicating promptly with healthcare providers and adjusting treatment plans, thereby maintaining higher medication adherence [46].

This study found that belief in the necessity of medication was a significant factor influencing the potential categories of medication adherence. Preliminary univariate analysis revealed significant differences in necessity belief scores among the three groups (*p* < 0.001). The good adherence group (median: 16.00) and the moderate adherence group (median: 14.00) both had higher scores than the poor adherence group (median: 10.00). After controlling for age and accessibility of medication, the Rank ANCOVA further confirmed these differences. Bonferroni post hoc tests indicated that significant differences existed not only between the poor adherence group and the other two groups (*p* < 0.001), but also between the good and moderate adherence groups (*p* < 0.001), suggesting a possible gradient relationship between belief in medication necessity and adherence level. Multinomial logistic regression analysis showed that, compared with the good adherence group, patients with lower necessity belief scores were more likely to be classified into the poor adherence group (OR = 0.376, 95% CI: 0.267 ~ 0.528, *p* < 0.001) and the moderate adherence group (OR = 0.708, 95% CI: 0.597 ~ 0.839, *p* < 0.001). Belief in the necessity of medication refers to an individual’s subjective cognition of the importance and necessity of drug therapy in preventing, controlling, or curing disease, reflecting the patient’s trust in the efficacy of the medication and the perceived intensity of the need for treatment [31]. Patients with stronger necessity beliefs can clearly recognize the key role of medication in controlling disease progression and preventing complications such as PTE and PTS [47]. When patients understand that taking medication is an essential means of restoring health, their motivation and proactive behaviors toward treatment are significantly enhanced, leading to better medication adherence [48].

This study found that concern about medication was a significant factor influencing the potential categories of medication adherence. Preliminary univariate analysis revealed significant differences in concern scores among the three groups (*p* = 0.018), with the poor adherence group showing a higher median score (15.00) than the good and moderate adherence groups (both 14.00). After controlling for age and accessibility of medication, the Rank ANCOVA confirmed an overall difference among the groups (*p* = 0.032). However, Bonferroni post hoc comparisons indicated that this difference was primarily driven by the contrast between the poor and good adherence groups (*p* = 0.034), while no significant differences were observed between the moderate group and the other two groups (both *p* > 0.05). Multinomial logistic regression analysis showed that, compared with the good adherence group, patients with higher concern scores were more likely to be classified into the poor adherence group (OR = 1.492, 95% CI: 1.156 ~ 1.926, *p* = 0.002).Concern about medication refers to patients’ worries or negative expectations regarding potential side effects, long-term use risks, or other adverse consequences of drug therapy, reflecting their perception of risks and attention to potential harms associated with treatment [31]. Patients with stronger medication concerns may reduce or discontinue medication use due to excessive fear of adverse reactions (e.g., gastrointestinal discomfort or bleeding). They may also question the efficacy or long-term safety of the medication, perceiving the potential risks as outweighing the benefits, thereby lowering their adherence to treatment [19].

The findings of this study provide important insights for the clinical management of patients with LEDVT, particularly regarding strategies to improve medication adherence. The results indicate that patients’ psychological and cognitive factors, such as perceived health competence, hope, activation, belief in the necessity of medication and concern about medication, play critical roles in determining medication adherence patterns. Healthcare workers should therefore move beyond purely pharmacological management and incorporate individualized psychological and behavioral assessments into routine care. For patients with low perceived health competence, targeted education and self-management training can enhance their confidence in managing treatment. Interventions designed to foster hope and patient activation, such as motivational interviewing and goal setting, may strengthen treatment engagement. Moreover, healthcare providers should actively address patients’ beliefs about medication by reinforcing its necessity while alleviating undue concerns about side effects through clear communication and shared decision-making. Integrating these psychosocial interventions into standard anticoagulation management could help improve medication adherence, enhance long-term therapeutic outcomes, and ultimately reduce recurrence and complications of LEDVT.

### Limitations

This study has the following limitations: (1) A key limitation is our inability to account for the phase and prescribed duration of anticoagulant therapy. As the standard treatment for post-surgical provoked VTE typically lasts 3–6 months, adherence behavior and psychological states are dynamic and likely differ between the initiation, middle, and conclusion of therapy. Our cross-sectional design, capturing data at a single time point, precludes the analysis of these temporal influences on the identified adherence profiles. (2) Other potential determinants of adherence, such as socioeconomic status, health literacy, out-of-pocket costs, and level of social support, were not measured. The absence of these variables means our model may not fully capture the complex web of factors influencing medication adherence. (3) Medication adherence and related psychological factors, such as perceived health competence, hope, activation, and medication beliefs, were primarily assessed through self-reports. This may introduce social desirability bias or recall bias, affecting data accuracy. Future studies should incorporate objective measures, such as electronic pillboxes, drug concentration monitoring, or physician assessments, to improve measurement reliability. (4) This study was conducted in four tertiary hospitals in Urumqi, all of which follow the current national guidelines and standardized protocols for anticoagulation management in China. This model of care, including the organization of healthcare services, approaches to patient education, and follow-up strategies, may differ from practices in other countries. Therefore, while our findings may be reasonably representative within the Chinese healthcare system, their generalizability at the international level should be interpreted with caution. Future multicenter studies conducted in other countries are warranted to further validate and extend these findings.

## Conclusion

This study employed a LPA to examine medication adherence and its influencing factors among patients with LEDVT in four tertiary hospitals in Urumqi, China. The results identified three distinct adherence profiles, including poor adherence group (44.99%), moderate adherence group (19.83%), and good adherence group (35.18%). Multinomial logistic regression analysis revealed that perceived health competence, hope, activation, belief in the necessity of medication, and concern about medication were key factors influencing the potential profiles of medication adherence among LEDVT patients. Healthcare workers should pay close attention to patients’ levels of medication adherence and develop targeted intervention strategies based on the characteristics and influencing factors of each adherence category. Such tailored interventions can effectively improve treatment adherence, reduce the risk of complications, and ultimately enhance patients’ quality of life and health outcomes.
